# Whole blood RNA signatures in leprosy patients identify reversal reactions before clinical onset: a prospective, multicenter study

**DOI:** 10.1038/s41598-019-54213-y

**Published:** 2019-11-29

**Authors:** Maria Tió-Coma, Anouk van Hooij, Kidist Bobosha, Jolien J. van der Ploeg-van Schip, Sayera Banu, Saraswoti Khadge, Pratibha Thapa, Chhatra B. Kunwar, Isabela M. Goulart, Yonas Bekele, Deanna A. Hagge, Milton O. Moraes, Rosane M. B. Teles, Roberta Olmo Pinheiro, Erik W. van Zwet, Jelle J. Goeman, Abraham Aseffa, Mariëlle C. Haks, Tom H. M. Ottenhoff, Robert L. Modlin, Annemieke Geluk

**Affiliations:** 1 Dept. of Infectious Diseases, Leiden University Medical Center, Leiden, The Netherlands; 2 Dept. Medical Statistics, Leiden University Medical Center, Leiden, The Netherlands; 3 Armauer Hansen Research Institute, Addis Ababa, Ethiopia; 4 International Center for Diarrhoeal Disease Research Bangladesh, Dhaka, Bangladesh; 5 Mycobacterial Research Laboratories, Anandaban Hospital, Kathmandu, Nepal; 6 National Reference Center for Sanitary Dermatology and Leprosy, Faculty of Medicine, Federal University of Uberlandia, Minas Gerais, Brazil; 7 Leprosy Laboratory, Oswaldo Cruz Institute - FIOCRUZ, Rio de Janeiro, Brazil; 8 Division of Dermatology, Department of Medicine, Immunology and Molecular Genetics, David Geffen School of Medicine at University of California (UCLA), Los Angeles, CA USA; 9 Department of Microbiology, Immunology and Molecular Genetics, David Geffen School of Medicine at University of California (UCLA), Los Angeles, CA USA

**Keywords:** Predictive markers, Biomarkers

## Abstract

Early diagnosis of leprosy is challenging, particularly its inflammatory reactions, the major cause of irreversible neuropathy in leprosy. Current diagnostics cannot identify which patients are at risk of developing reactions. This study assessed blood RNA expression levels as potential biomarkers for leprosy. Prospective cohorts of newly diagnosed leprosy patients, including reactions, and healthy controls were recruited in Bangladesh, Brazil, Ethiopia and Nepal. RNA expression in 1,090 whole blood samples was determined for 103 target genes for innate and adaptive immune profiling by dual color Reverse-Transcription Multiplex Ligation-dependent Probe Amplification (dcRT-MLPA) followed by cluster analysis. We identified transcriptomic biomarkers associated with leprosy disease, different leprosy phenotypes as well as high exposure to *Mycobacterium leprae* which respectively allow improved diagnosis and classification of leprosy patients and detection of infection. Importantly, a transcriptomic signature of risk for reversal reactions consisting of five genes (*CCL2, CD8A, IL2, IL15* and *MARCO*) was identified based on cross-sectional comparison of RNA expression. In addition, intra-individual longitudinal analyses of leprosy patients before, during and after treatment of reversal reactions, indicated that several IFN-induced genes increased significantly at onset of reaction whereas *IL15* decreased. This multi-site study, situated in four leprosy endemic areas, demonstrates the potential of host transcriptomic biomarkers as correlates of risk for leprosy. Importantly, a prospective five-gene signature for reversal reactions could predict reversal reactions at least 2 weeks before onset. Thus, transcriptomic biomarkers provide promise for early detection of these acute inflammatory episodes and thereby help prevent permanent neuropathy and disability in leprosy patients.

## Introduction

Leprosy is a chronic infectious disease caused by *Mycobacterium leprae*, a bacillus with tropism for skin and peripheral nerves. Despite decades of programs using multidrug therapy (MDT), leprosy remains persistently endemic or re-emerging in some regions where it predominantly affects poor and marginalized people. A featuring aspect regarding leprosy diagnosis is the plateauing annual new case detection rates of roughly 200,000 worldwide^1^. The pathology of leprosy is complex as it presents as a spectral disease in which immunity against *M. leprae* matches the clinical manifestations after infection with the bacterium. At one pole of the spectrum, the disease manifests as tuberculoid leprosy (TT), characterized by strong pro-inflammatory cellular immunity including Th1 and Th17 cells^2,3^, granuloma formation and elimination of bacteria. At the other pole, lepromatous leprosy (LL) is characterized by humoral immunity against *M. leprae* along with Th2 cells but almost no protective cell mediated immunity, allowing accumulation of high numbers of bacilli around foamy macrophages^4–8^. Nonetheless, the majority of individuals present unstable borderline phenotypes (BT, BB and BL) between the two poles^5^.

A major challenge in leprosy control is the prevention of permanent disability due to nerve damage. Although leprosy is curable by MDT, nerve damage cannot always be avoided. Dynamic and unpredictable episodes of increased inflammation, leprosy reactions, can occur before, during and even after treatment, with a higher likelihood to occur in adults than in children^9,10^. These immunological complications are the principal cause of leprosy-associated irreversible neuropathy and are experienced by 30–50% of leprosy patients one or more times, mostly in the unstable borderline lepromatous patients with substantial bacterial loads^11^. Two types of reactions are recognized: reversal reactions or type 1 (RR) and erythema nodosum leprosum (ENL). RRs are caused by changes in the host immune response against *M. leprae* which is upgrading from borderline to the TT pole characterized by an enhanced cell-mediated immunity, inflammation^12,13^. These reactions can occur spontaneously but are also linked to shifts from Th2 to Th1, e.g. occurring during anti-helminth treatment of co-infected leprosy patients^14–17^, HIV highly active antiretroviral therapy (HAART) and at the end of extensive anti-TNF-α therapy^10,13^ and even BCG vaccination^18^.

Prompt diagnosis and treatment of reactions significantly favors successful recovery^9,19^. Unfortunately, reactions are often late- or misdiagnosed, in part due to decreased expertise within integrated health services^19^ which urges the need for new diagnostic tools. Delays in diagnosis of reactions directly translate into negative clinical outcomes, as associated neuropathy not properly diagnosed or treated within the first 6 months of symptoms will likely become permanent^20^ alongside the disabilities it may later initiate via recurrent ulcers and other related pathologies^21^. Despite recent scientific progress with respect to complement^22,23^ and serum-proteins, particularly CXCL10 (IP-10), as biomarkers associated with onset of reactions^15–17,24–26^, discovery of accurate, clinically useful prognostic biomarkers remains elusive, leaving early diagnosis of reactions a currently unmet need.

Since host transcriptomic biomarkers reflect early stages of or ongoing biological processes, they have been widely used to profile the host transcriptome for diagnostics of tuberculosis (TB)^27–30^. Moreover, multicomponent host biomarker signatures have been described that predict development of disease in retro- and prospective cohorts^31,32^. In this respect dual color Reverse-Transcription Multiplex Ligation-dependent Probe Amplification (dcRT-MLPA) has proven to be a valuable tool for monitoring gene expression profiles in large cohorts^29,33^. Techniques such as RNA-Seq and microarray are costly, technically challenging and require high RNA concentrations which limits their application for large cohorts. Therefore, a selection of genes related to immune-mediated inflammatory pathways, which play a role in the immunopathology of leprosy can be assessed by dcRT-MLPA^29,34^.

Many reactions occur during MDT, with the highest rates reported within the first 6 months of treatment^11,19,35^. To identify transcriptomic signatures for applications to surveillance of leprosy reactions, whole blood RNA of leprosy patients was monitored during MDT. To accommodate worldwide applicability, this study was executed in four prospective cohorts in Asia, Africa and South America. Improved knowledge on longitudinal fluctuations of RNA expression associated with reactions will promote identification of patients with imminent reactions leading to timely interventions that can impact nerve damage in affected individuals.

## Materials and Methods

### Participants

Patients and controls were recruited on a voluntary basis between February 2008 and March 2015 (Table 1) in four leprosy endemic populations: in Bangladesh (International Centre for Diarrhoeal Disease Research Bangladesh, Dhaka), Brazil (National Reference Centre for Sanitary Dermatology and Leprosy, Uberlandia and Leprosy Laboratory, Oswaldo Cruz Institute, Oswaldo Cruz Foundation (FIOCRUZ), Rio de Janeiro), Ethiopia (ALERT hospital and Health Centre, Addis Ababa) and Nepal (Mycobacterial Research Laboratories, Kathmandu). Leprosy was diagnosed based on clinical, bacteriological and histological observations and classified by skin biopsies according to Ridley-Jopling^36^. Clinical monitoring for reactions was performed during monthly clinic visits. Participant information was collected with emphasis on standardizing data collection and definition of reactions between all cohorts^37,38^. Endemic controls (EC) were living in the same area without known contact with leprosy or TB patients and were assessed for the absence of clinical signs and symptoms of leprosy and TB. Staff of leprosy or TB clinics and laboratory staff were excluded. Healthy household contacts (HHC) were defined as adults living in the same household as leprosy patients for at least the preceding six months.

**Table 1:** Samples of participants included in cross-sectional analysis.

Site	Category^a^	LR^b^	Timepoint^c^	Number
**Bangladesh**	EC	na	na	61
	BB/BL/LL (MB) no LR t = 0	no LR	t = 0	62
	BB/BL/LL (MB) no LR t = end	no LR	t = end	26
	TT/BT (PB) no LR t = 0	no LR	t = 0	36
	TT/BT (PB) no LR t = end	no LR	t = end	9
	BB/BL/LL (MB) RR t = 0	RR	t = 0	5
	BB/BL/LL (MB) RR t = x	RR	t = x	30
	BB/BL/LL (MB) RR t = end	RR	t = end	42
	TT/BT (PB) RR	RR	t = 0 or = x, or = end	9
	ENL/Neuritis	ENL/Neuritis	t = 0 or = x, or = end	11/0
	HHC	na	na	38
**Brazil**	EC	na	na	46
	BB/BL/LL (MB) no LR t = 0	no LR	t = 0	26
	BB/BL/LL (MB) no LR t = end	no LR	t = end	20
	TT/BT (PB) no LR t = 0	no LR	t = 0	52
	TT/BT (PB) no LR t = end	no LR	t = end	38
	BB/BL/LL (MB) RR t = 0	RR	t = 0	17
	BB/BL/LL (MB) RR t = x	RR	t = x	20
	BB/BL/LL (MB) RR t = end	RR	t = end	20
	TT/BT (PB) RR	RR	t = 0 or = x, or = end	28
	ENL or Neuritis	ENL/Neuritis	t = 0 or = x, or = end	22/18
	HHC	na	na	14
**Ethiopia**	EC	na	na	51
	BB/BL/LL (MB) no LR t = 0	no LR	t = 0	83
	BB/BL/LL (MB) no LR t = end	no LR	t = end	9
	TT/BT (PB) no LR t = 0	no LR	t = 0	16
	TT/BT (PB) no LR t = end	no LR	t = end	1
	BB/BL/LL (MB) RR t = 0	RR	t = 0	2
	BB/BL/LL (MB) RR t = x	RR	t = x	36
	BB/BL/LL (MB) RR t = end	RR	t = end	12
	TT/BT (PB) RR	RR	t = 0 or = x, or = end	6
	ENL or Neuritis	ENL/Neuritis	t = 0 or = x, or = end	11/1
	HHC	na	na	33
**Nepal**	EC	na	na	42
	BB/BL/LL (MB) no LR t = 0	no LR	t = 0	14
	BB/BL/LL (MB) no LR t = end	no LR	t = end	5
	TT/BT (PB) no LR t = 0	no LR	t = 0	19
	TT/BT (PB) no LR t = end	no LR	t = end	8
	BB/BL/LL (MB) RR t = 0	RR	t = 0	6
	BB/BL/LL (MB) RR t = x	RR	t = x	12
	BB/BL/LL (MB) RR t = end	RR	t = end	7
	TT/BT (PB) RR	RR	t = 0 or = x, or = end	29
	ENL or Neuritis	ENL/Neuritis	t = 0 or = x, or = end	2/0
	HHC	na	na	6
**Netherlands**	NEC first time point	na	na	19
	NEC second time points	na	na	10
			**total**	**1090**

^a^EC: endemic control; BB/BL/LL: borderline borderline/borderline lepromatous/lepromatous leprosy; TT/BT: tuberculoid leprosy/borderline tuberculoid leprosy; ^b^LR: leprosy reaction; RR: reversal reaction; NEC: non-endemic control; na: not applicable; ^c^t = 0: time point of enrolment before initiation of multidrug (MDT) therapy; t = x: time point of LR; t = end: time point of completion of MDT and/or steroid therapy.

### Recruitment

Newly diagnosed, untreated leprosy patients without clinical reactions were enrolled and blood was drawn before initiation of MDT(t = 0) as previously described^15^. Patients with reactions within initiation of three months of therapy were excluded. Patients were often diagnosed with RR at first clinic visits, leading to a low frequency of untreated cases without RR at their first visits that subsequently developed RR during this study. If patients presented with reactions after more than three months of MDT, blood was drawn again before initiation of anti-reactional therapy (t = x). Patients diagnosed with RR at their first clinic visits were also recruited (t = x) but blood was collected after completion of MDT and/or after steroid therapy (t = end). For patients with RR this was done at least one month after completion of steroid therapy. Patients were assessed for absence of reactions one year after t = end. For patients showing clinical signs of reactions within three months after t = end, this time point was excluded from analyses. Thus, analyses included two samples of each patient without reactions [before (t = 0) and after treatment (t = end)] and three of each patient who developed RR [in the absence of clinical signs of reactions, ≥2 months before RR diagnosis (t = 0); at RR diagnosis, before steroid-treatment (t = x); after RR, at least one month after ending steroid-treatment (t = end)]. Patients with leprosy relapse and pure neural leprosy were excluded from the analysis.

### Ethics

This study was performed according to the Helsinki Declaration. Written informed consent was obtained before enrolment. Patients received treatment according to national guidelines. Ethical approval of the study-protocol was obtained through Ethical Review Committee of ICDDR,B (#PR-10032; #PR-2007-069); Brazilian National Council of Ethics in Research (CONEP) and the Fiocruz Ethical research Council CEP (# 555/10) or UFU Research Ethics Committee (#499/08); National Health Research Ethical Review committee Ethiopia (NERC # RDHE/127-83/08); Nepal Health Research Council (NHRC #751).

### RNA isolation

RNA from PAXgene tubes was extracted using PAXgene Blood RNA kits (BD Biosciences, Franklin Lakes, NJ) according to the manufacturers’ protocol. RNA yield was determined by a NanoDrop ND-1000 spectrophotometer (NanoDrop Technologies, Wilmington, DE).

### Dual color reverse-transcription multiplex ligation-dependent probe amplification (dcRT-MLPA) assays

dcRT-MLPA assay was performed as previously described^33,39^. In short, for each target-specific sequence, a specific RT primer was designed located downstream of the half-probe target sequences (Sigma-Aldrich, Saint Louis, MO). RNA (2.5 μl of a 50 ng/μl solution) was reverse transcribed with 1x MMLV reverse transcriptase buffer, dNTPs (0.4 mM of each nucleotide), and 80 nM of the target-specific RT primers in a final volume of 4.5 μl. After heating for 1 min to 80 °C and incubation for 5 min at 45 °C, 30U MMLV reverse transcriptase (Promega, Madison, WI) was added and incubated for 15 min at 37 °C before heat inactivation of the enzyme for 2 min at 98 °C. Subsequently, half-probes (6 nM) were added to the reaction, heat denatured for 1 min at 95 °C followed by hybridization for 16 h at 60 °C. Ligation of the annealed half-probes was performed for 15 min at 54 °C by ligase-65 followed by heat inactivation for 5 min at 98 °C. Ligation products were amplified by PCR. Thermal cycling conditions encompassed: 33 cycles of 30 s/95 °C, 30 s/58 °C, and 60 s/72 °C, followed by 1 cycle of 20 min/72 °C. PCR products were diluted 1:10 in HiDi formamide containing 400 HD ROX size standards and analyzed on an Applied Biosystems 3730 capillary sequencer in GeneScan mode (Applied Biosystems, Foster City, CA). MLPA reagents were from MRC-Holland (Amsterdam, The Netherlands).

Data were analyzed using GeneMapper software 5 (Applied Biosystems). The areas of each assigned peak (in arbitrary units) were exported for further analysis in R-Project, normalized to *GAPDH* and log2 transformed. Signals below the threshold value for noise cut-off (peak area ≤ 7.644) were assigned the threshold value for noise cut-off. Pathway analysis was performed using Ingenuity Pathway Analysis (Qiagen, Hildern, Germany).

### Statistical analyses dcRT-MLPA

Wilcoxon signed-rank test was applied for paired samples. For cross-sectional comparisons of different test groups at comparable time points, a Mann–Whitney test was used. P-values were corrected for multiple comparisons using the Benjamini-Hochberg method^40^. To classify BL/LL patients according to their likelihood to develop RR a logistic regression model was fitted with RR (yes/no) as outcome (dependent variable), and gene expression values at time t = 0 as covariates (risk factors). Genes were grouped based on correlation of their expression using a hierarchical clustering analysis (average linkage) based on absolute correlation difference. The global test (version 5.32.0)^41^ in R (version 3.4.1) was performed on these groups. Genes that were significantly differentially expressed between the test groups (inheritance <0.05) after multiple testing correction, constituted a biomarker signature for prediction of RR. To avoid overfitting, the biomarker signature was evaluated using a leave-one-out cross-validation (LOOCV): during the training of the model, one subsample (n-1) was reserved in each iteration for evaluation of the accuracy of the model on a sample excluded from the training set. The gene selection for the predictive signature was redone at every fold. The predictive biomarker signature was thus assessed in observations, which were not used to build the model. To evaluate the risk of developing RR, the cut-off for gene expression was determined based on the Youden Index. A score of 0 or 1*(weight of gene) was given per gene based on the association to RR (as indicated by the cut-off). The weight of gene was based on the results from the global test. All the scores from the significant genes were added and divided by the sum of weights to calculate the risk to develop RR. Area Under the Receiver Operating Characteristic curve (ROC-AUC) was calculated using GraphPad Prism (version 7.02).

## Results

### Prospective cohorts

To identify correlates of risk (CoR) for leprosy and RR, blood of 1,090 samples was obtained longitudinally in Bangladesh, Brazil, Ethiopia and Nepal (Tables 1 and 2).

**Table 2:** Demographics and clinical characteristics of reactional patients recruited longitudinally^§^.

#	area	RJ	LR	BI*	PGL-I*	sex	age*
1	Bangladesh	BL	RR	2+	0.95	male	36
2	Bangladesh	BL	RR	2+	0.27	female	39
3	Bangladesh	BL	RR	0	1.82	male	36
4	Bangladesh	BL	RR	2+	1.17	male	32
5	Brazil	BL	RR	3.2+	0,43	male	35
6	Brazil	BL	RR	2.42+	0.07	male	29
7	Brazil	BL	RR	4.28+	1.02	female	42
8	Nepal	BT	RR	0.25+	0.07	female	40
9	Ethiopia	BB	RR	0	0.15	male	33
10	Netherlands	BL	RR	5+	1.83	male	17

^§^Blood samples were collected from 10 patients at 3 time points: at diagnosis of leprosy in the absence of any clinical signs of reactions, at diagnosis of reactions and after treatment; * at recruitment before treatment.

RJ: Ridley Joplin classification; LR: Leprosy Reaction; RR: reversal reaction.

BI: bacterial index; PGL-I: OD at 450 nm in anti-PGL-I antibody ELISA (threshold for positivity: OD_450_ = 0.2).

### Leprosy-specific RNA-profiles

We first analyzed gene expression in *ex vivo* blood samples of newly diagnosed leprosy patients (irrespective of classification) without reactions (n = 359) from Bangladesh, Brazil, Ethiopia and Nepal compared to EC from these regions (n = 200). To this end, 103 target genes associated with innate and adaptive immunity^29,33,39^ were analyzed by dcRT-MLPA (Table S1). A substantial variety (36 genes) was observed to significantly differ between patients and EC at all sites. Expression of 13 genes was upregulated and 23 genes were downregulated in patients compared to EC (Table S2). When comparing leprosy patients to HHC (Table S3), 16 genes showed significantly different expression for leprosy patients (increased: *FCGR1A*, *IL6*, *IL15*, *LRKK2*, *MBP*, *MSR1*, *PACRGv1*, *TLR1*, *TLR4*; decreased: *CAMTA*, *CD3E*, *CTLA4*, *CXCL13*, *GATA3*, *LAG3*, *TFGB*). Importantly, whilst most of these genes were also differently expressed in leprosy patients compared to EC, *MBP*, *MSR1, TLR1, CAMTA, CXCL13* and *TFGB* were differentially expressed in leprosy patients exclusively when compared to HHC. Such genes are potential CoR for leprosy in contacts of leprosy patients who are highly exposed to *M. leprae*. Being part of innate/adaptive and macrophage signaling pathways^42^, these genes, are not restricted to leprosy but are also relevant in rheumatoid arthritis and Crohn’s disease (Table S4).

### RNA-profiles for leprosy classification

**Figure 1:**
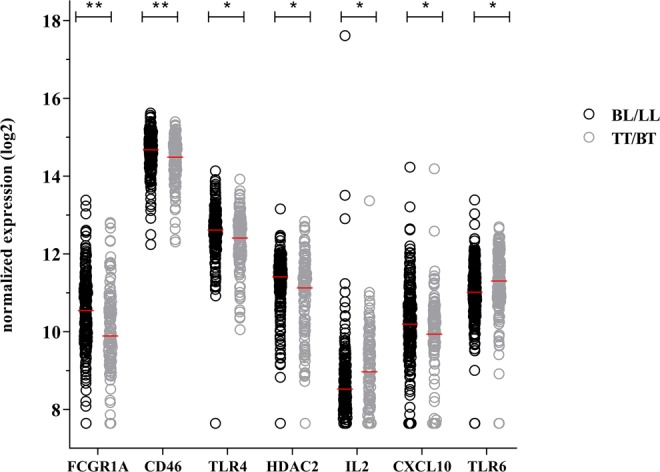
Transcriptional profiles of BL/LL and TT/BT patients without reactions. Gene expression levels of 103 target genes were assessed by dual-color RT-MLPA performed on *ex vivo* RNA isolated from whole blood of newly diagnosed, untreated BL/LL (n = 228; black circles) and TT/BT (n = 131; grey circles) leprosy patients without reactions from Bangladesh, Brazil, Ethiopia and Nepal. Log2-transformations of peak areas (normalized to the housekeeping gene *GAPDH*) of genes that were significantly differentially expressed between BL/LL and TT/BT are shown on the *y*-axis. Raw p-values were calculated using the Mann–Whitney test and adjusted for multiple comparisons using the Benjamini-Hochberg correction^40^. *adjusted p-values < 0.05; **adjusted p-values < 0.01; (see Table S5).

To identify genes applicable for classification of different types of leprosy, the 103 target genes (Table S1) were assessed similarly by dcRT-MLPA in blood from newly diagnosed, untreated BL/LL (n = 228) as well as TT/BT (n = 131) leprosy patients from the four-different leprosy endemic populations. After correction for multiple comparisons^40^ seven genes remained significantly different. Expression levels of *IL2* and *TLR6* were increased in TT/BT compared to BL/LL whereas *CD46, CXCL10, FCGR1A, HDAC2* and *TLR4* expression levels were increased in BL/LL (Fig. 1; Table S5).

### RNA-profiles associated with exposure to *M. leprae*

**Figure 2:**
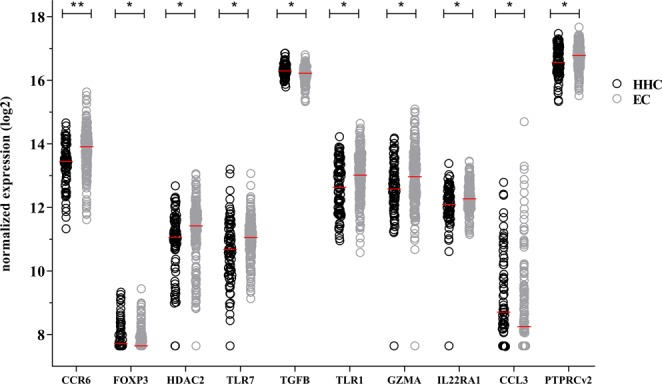
Transcriptional profiles of healthy household contacts and endemic controls. Gene expression levels of 103 target genes were assessed by dual-color RT-MLPA performed on *ex vivo* RNA isolated from whole blood of healthy household contacts (HHC; n = 83; black circles) and endemic controls (EC; n = 200; grey circles) from Bangladesh, Brazil, Ethiopia and Nepal. Log2-transformations of peak areas (normalized to *GAPDH*) of genes significantly differentially expressed between HHC and EC are shown on the *y*-axis. Raw p-values were calculated using the Mann–Whitney test and adjusted for multiple comparisons using the Benjamini-Hochberg correction^40^. *adjusted p-values < 0.05; **adjusted p-values < 0.01; (see Table S6).

Since HHC of leprosy patients have a higher risk to develop leprosy than the general population in an endemic area^43^, biomarker profiles indicating this risk could help decision making on who needs preventive antibiotic treatment^44^. These genes represent potential transcriptomic tools to identify individuals substantially exposed to *M. leprae*. Thus, RNA-expression profiles of HHC (n = 83) were compared to EC (n = 200) (Table S6). We identified 10 differentially expressed genes with either significantly higher expression in HHC (Fig. 2; *FOXP3, TGFB* and *CCL3*) or in EC (*CCR6, GZMA, HDAC2, IL22RA1, PTPRCv2, TLR1* and *TLR7*).

### Transcriptomic risk factors for development of RR

**Figure 3:**
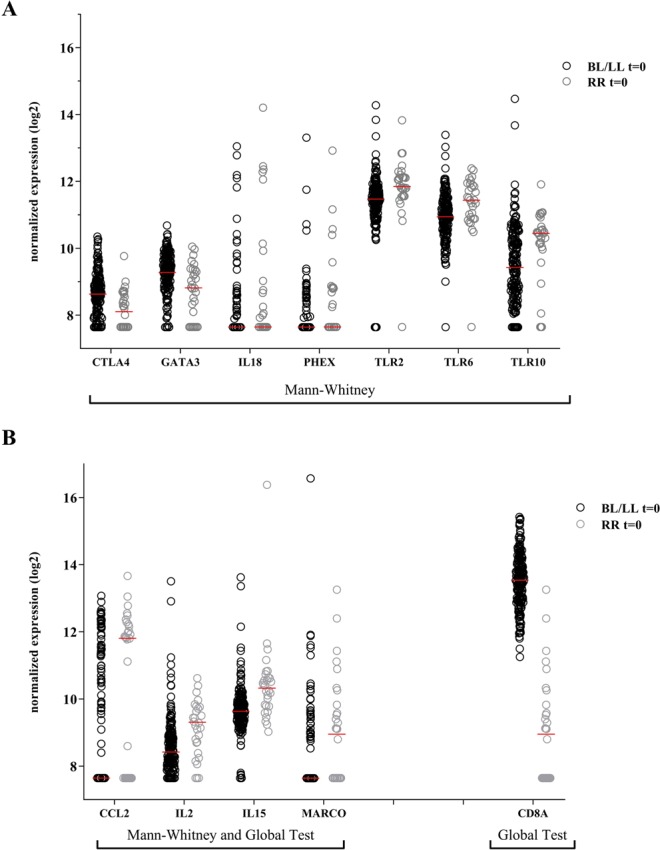
Identification of biomarker risk signature for developing reversal reactions. Gene expression levels of 103 target genes were assessed by dual-color RT-MLPA performed on *ex vivo* RNA isolated from whole blood from BL/LL patients who developed reversal reactions (RR) at least two months later during the study (n = 30; grey circles) and BL/LL who did not develop reactions (n = 184; black circles) from Bangladesh, Brazil, Ethiopia and Nepal. Samples were analyzed at t = 0: in the absence of clinical signs of reactions. Log2-transformations of peak areas (normalized for *GAPDH* expression) of genes with significantly different expression between both groups (at t = 0) are shown on the *y*-axis. (**A**) Genes with a significant (p-value < 0.05) different expression only using the Mann-Whitney test are show. P-values were adjusted for multiple comparisons using the Benjamini-Hochberg correction^40^ (see Table S7). (**B**) Genes with a significant different expression in the global test and Mann-Whitney or the global test only are shown.

To assess whether RNA-expression levels can be used as a predictive tool for reactions during MDT, cross-sectional comparison of gene expression levels was performed for samples at the time of leprosy diagnosis in the absence of clinical signs of reactions (t = 0). Gene expression from BL/LL patients who developed RR ≥ 2 months later in the study (n = 30) was compared to BL/LL patients who did not develop reactions at all (n = 184) using the Mann–Whitney test. Transcriptomic profiles of the two groups of BL/LL patients resulted in a decreased expression of *CTLA4* and *GATA3* at diagnosis in patients who would later develop RR (Fig. 3), whereas nine genes (*CCL2, IL2, IL15, IL18, MARCO, PHEX, TLR2, TLR6* and *TLR10*) were significantly increased (Fig. 3; Table S7).

**Figure 4:**
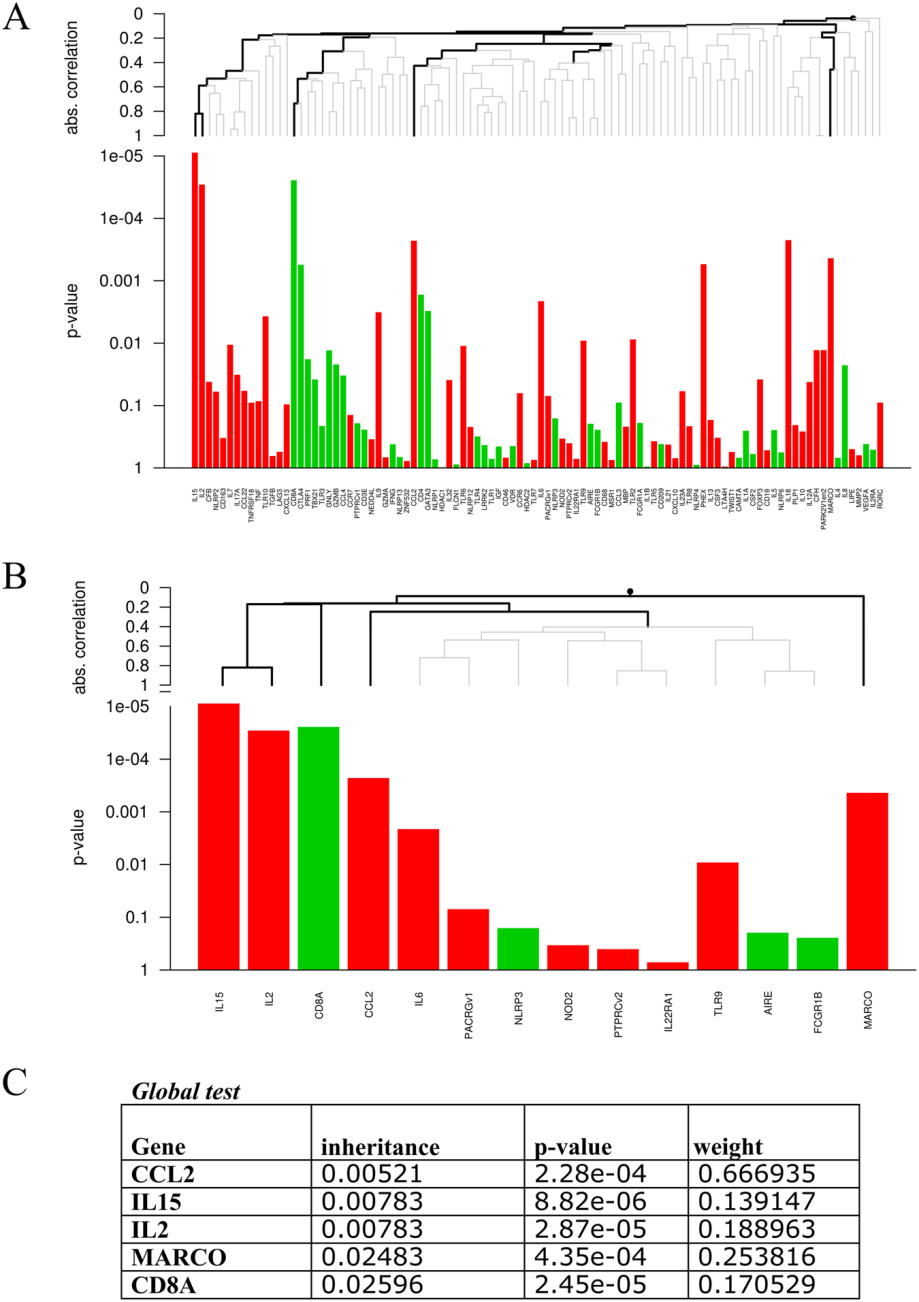
Identification of a minimal biomarker risk signature for developing reversal reactions. Biomarker signature to assess the risk of BL/LL patients to develop reversal reactions (RR). Gene expression data obtained by dual-color RT-MLPA of RNA isolated from whole blood of BL/LL patients from Bangladesh, Brazil, Ethiopia and Nepal at t = 0 were analyzed using the global test cluster analysis^41^. The global test is a cluster analysis based on absolute correlation difference and average linkage developed for data sets in which many covariates (or features) have been measured for the same subjects, together with a response variable. Graphs (**A,B**) indicate genes that are higher expressed in future RR patients (red) or in non-reactional BL/LL patients (green). In (**A**) all genes analyzed are shown and in (**B**) only significant branches are shown. Table (**C**) shows values for the 5 genes that were statistically significant after correction for multiple testing (inheritance <0.05), representing the output signature of the global test shrinkage model.

Next, we also determined the biomarker signature with the least number of genes and the highest discriminatory power to categorize BL/LL patients according to their likelihood to develop RR using the global test^41^ (Fig. 4; p = 1.28*10^−5^). Four genes (*CCL2, IL2, IL15* and *MARCO*) remained significantly (inheritance <0.05) associated with occurrence of RR at the time of recruitment, whereas *CD8A* was negatively associated with RR (Fig. 3B). Although expression of CD8A was not statistically significant using Mann–Whitney, it contributed significantly to the transcriptomic global test-signature. From the five genes *CCL2* contributed most to the model (Fig. 4B).

To evaluate the prediction a LOOCV was performed in which in every iteration a subsample (n = 1) was excluded in the global test to evaluate the model performance on unseen samples. The overall classifying ability of the biomarker-signature is indicated as a ROC-AUC = 0.80 (Fig. S1). Thus, transcriptomic profiles can prospectively differentiate, at the time of leprosy diagnosis in the absence of any clinical symptoms of reactions, patients who will develop RR.

### Longitudinal transcriptomic changes: monitoring RR onset and treatment

Cross-sectional analysis of gene expression at different time points amongst patients with RR showed only a significant increase in IL10 at RR compared to before RR (Supplementary results; Tables S8 and S9; Figs. S2 and S3).

**Figure 5:**
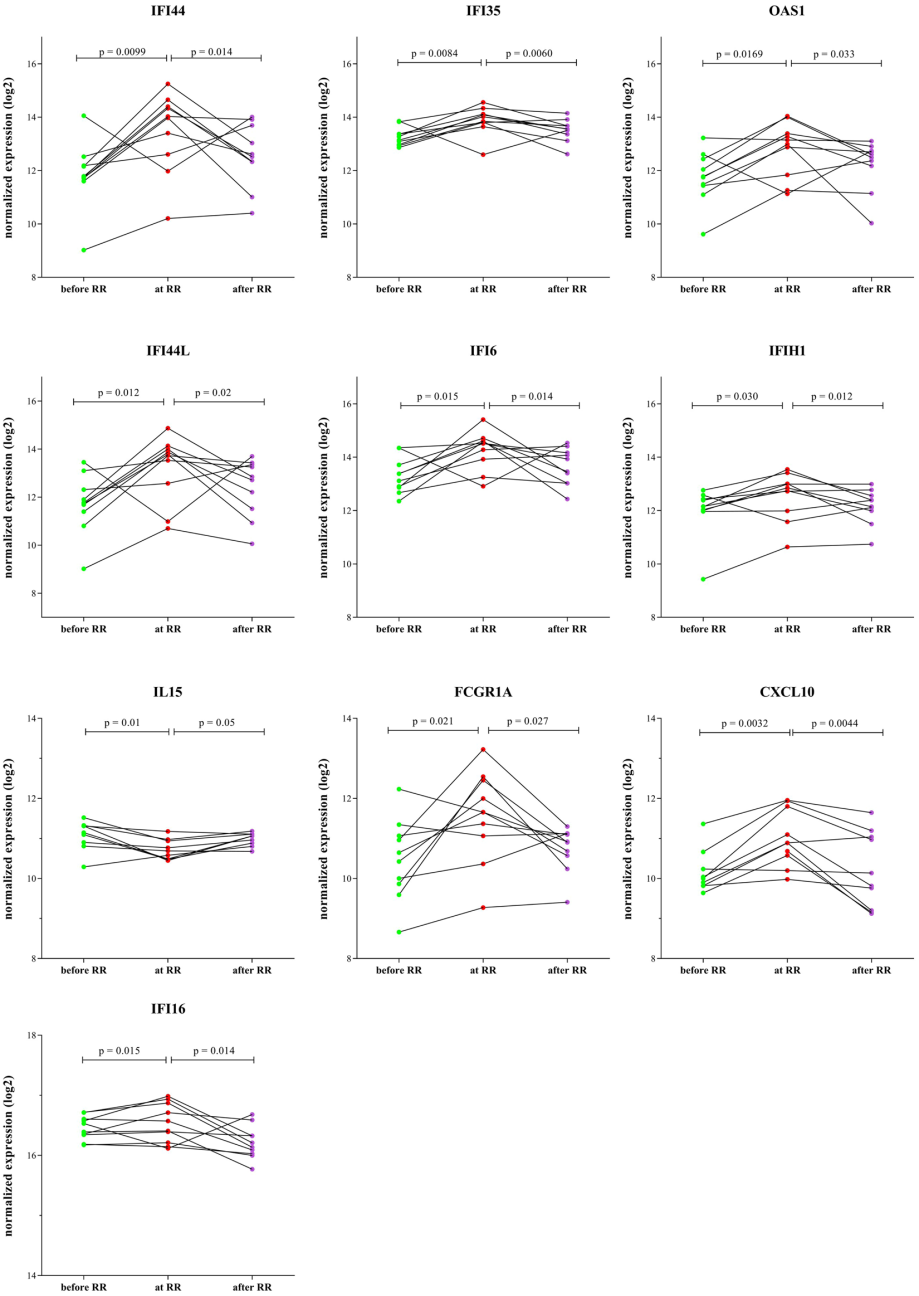
Intra-individual longitudinal expression of patients who developed RR. Direct *ex vivo* RNA expression values were assessed by dual-color RT-MLPA on whole blood of 10 leprosy patients who developed RR during this study. Blood was analyzed at three time points: in the absence of any clinical signs of reactions and at least two months before RR (t = 0), at RR diagnosis before steroids (t = x) or after MDT and at least one month after end of steroids, in the absence of reactions (t = end). Log2-transformations of peak areas (normalized for *GAPDH* expression) are shown on the *y*-axis. Wilcoxon signed-rank test was performed. Genes with a significant difference (p-value < 0.05) in expression between before RR-at RR and at RR-after RR are shown.

Since RNA expression levels of genes may vary over time, RNA expression was also assessed longitudinally at three time points in whole blood of 10 leprosy patients (Table 2) developing RR during the study (Figs. 5, S4 and S5). This included besides 103 immune-associated genes, 38 IFN-induced genes (Table S1) previously identified as markers for mycobacterial disease^45–49^. Expression of ten genes (*CXCL10, FCGR1A, IFI16, IFI44, IFI35, IFI44L, IFI6, IFIH1, IL15 and OAS1*) significantly differed when comparing time points before RR vs at RR as well as at RR vs after RR. All genes except *IL15* consistently increased with development of RR, and normalized after treatment, whereas *IL15* decreased at onset of RR (Fig. 5). Most of the genes identified longitudinally are directly connected to the genes identified as predictive markers before clinical symptoms of RR (Fig. S6).

Five genes (*IL10, PRF1, CCL2, CCL3* and *BMP6*) were significantly different only at onset of RR compared to before RR (Fig. S4), while nine (*IFITM3, IFIT3, GBP5, GBP1, GBP2, OAS3, STAT1, STAT2* and *TAP1*) only displayed a significant effect upon treatment (Fig. S5).

These data indicate that onset of RR can be monitored, based on differential expression of inflammatory genes in whole blood, allowing early detection and subsequent treatment of RR. Thus, helping to reduce irreversible nerve damage and associated disabilities.

## Discussion

In the current state of leprosy elimination, reactions persist as a major problem since patients remain at risk due to *M. leprae* antigens that can persist for years post-MDT. RRs can occur at any time during, before or after MDT^12^ and although several factors have been associated with reactions, the underlying mechanism is not completely known^10^. Genetic susceptibility to reactions but also treatment for other diseases or co-morbidities have been suggested to play a role by causing an immunological shift from Th2 to Th1^10,13–17^. In addition, Th17 cells have been proposed to play a role in RR pathogenesis as well^50^. Still, no validated biomarker signature is available at the moment. The implementation of diagnostics tests for reactions in leprosy health care could make significant differences in clinical outcomes and help reduce nerve damage. Transcriptomic biomarker signatures provide indications as to how immune responses are oriented and instructed to develop reactions^31,32^. Due to the available samples set we have focused on the analysis of biomarkers for RR; biomarkers for CoR of leprosy, leprosy classification and *M. leprae* exposure have also been investigated.

This is the first multi-site, prospective study analyzing unstimulated whole blood-derived RNA expression profiles of four endemic populations using dcRT-MLPA, a focused gene expression profiling platform for monitoring gene expression in large cohorts^29,34^. We showed that host RNA expression levels discriminate various stages of disease thereby offering potential for leprosy diagnostics. Since differentiating high exposure to *M. leprae* and early leprosy is difficult using serum proteins^51^, the here identified transcriptomic biomarkers provide potential to identify cases among contacts in high endemic regions. A limitation of the current study is the lack of follow-up of HHC in order to validate the identified biomarkers of *M. leprae* infection as CoR for leprosy. However, this is currently addressed in ongoing studies. Moreover, validation in an independent cohort will provide more evidence on whether transcriptomic signatures may be applied to guide prophylactic strategies by discriminating contacts highly exposed to *M. leprae* and at risk of developing disease.

We have identified genes differentially expressed between leprosy patients and EC or HHC. Whilst most of the genes overlap between the two comparisons, some genes discriminate leprosy patients from HHC exclusively (*MBP*, *MSR1, TLR1, CAMTA, CXCL13* and *TFGB*). Thus, these genes could be correlated to development of disease. Additionally, we observed that a classification of leprosy patients is possible using transcriptomics since a set of genes were increased in BL/LL patients (*CD46, CXCL10, FCGR1A, HDAC2* and *TLR4*) and TT/BT showed a higher expression of *IL2* and *TLR6*.

Importantly, we identified a five-gene CoR signature (*CCL2, CD8A, IL2, IL15*, *MARCO*) for RR differentiating those developing RR ≥ 2 months prior to clinical symptoms. *CCL2*, was downregulated in leprosy patients without reactions compared to EC, in line with lower *CCL2* expression observed in nerves of leprosy patients compared to patients with non-leprous peripheral neuropathy^52^. We observed a higher expression of *CCL2* in future RR patients compared to patients who did not develop RR, as well as a longitudinal increase during RR onset. Similarly, a Brazilian study analysing 90 immune related genes, showed that *CCL2* had the highest fold change in expression levels between leprosy patients with and without RR^53^ and increased *CCL2* expression in RR patients has also been described in other studies^35,54^. *CCL2* is associated with excessive deposit of extracellular matrix and macrophage recruitment^55^ which can be due to an increase of *M. leprae* antigens presented to the immune system after MDT. This leads to activation of pro-inflammatory cytokines and attraction of CD4^+^ T cells as confirmed by the similarly increased *IL2* expression in future RR patients. Upregulation of *IL2* and *IL15*, as well as downregulation of *CTLA4* and *GATA3* decreases regulation of Th2 and the lack of regulation leads to exacerbation of Th1, which is common in RR. Higher expression of *MARCO* in future RR patients is also in line with increased antigen presence. Differences in the expression of this scavenger receptor were also identified in several other diseases such as giant cell arteritis^56^, enthesitis-related arthritis^57^ and lupus^58^ suggesting a general role for antigenic triggers in respective disease etiology. *IL15* which encodes a cytokine important for cytotoxic T-cell proliferation and increases *GZMB* expression^59^, was higher expressed in future RR patients, but decreased during RR in the longitudinal analysis. Upregulation of *IL15* in patients who will develop RR may lead to an increase of cytotoxic T-cells and tissue destruction. *CD8A* is found on cytotoxic T lymphocytes, macrophages and dendritic cells leading to tissue damage in leprosy^60^. Even though we did not find significant difference in the expression before and at RR, previous longitudinal analysis showed increased expression at RR onset^39^. Thus, the decreased expression of *CD8A* as observed here in future RR patients when they still lack reactional symptoms (cross-sectional comparison) may indicate the number of CD8^+^ cells like cytotoxic T cells are increasing during RR development.

To evaluate the role of reaction-associated biomarkers as etiological and early disease prediction targets, temporal associations are implicit to indicate the utility of novel biomarkers for application in diagnostic tests. In this multi-site study, RNA expression was therefore also analyzed longitudinally: before, during reaction and after treatment. IFN-induced genes (*IFI44, IFI35, IFI44L, IFI6, IFIH1, CXCL10 and FCGR1A*) showed high expression at RR which decreased during reactional treatment; whereas *IL15* expression decreased at RR. These biomarkers corresponding with increased inflammation can be useful when monitoring patients during their monthly dose of rifampicin helping to timely detect incipient RR. However, larger longitudinal cohorts should be studied prospectively to confirm our findings.

Previously, using longitudinal analysis of blood and skin samples of one leprosy patient who developed RR, a candidate blood-derived CoR for RR was identified composed of genetic host factors associated with T-cell cytotoxicity, regulation, vasculogenogenesis and IFN-signaling^15^. An interferon-dominant signature has also been identified for TB, first in 2010^45^ and in multiple subsequent studies^47,49,61^. In line with our findings, higher levels of *CXCL10* have also been reported in association with episodes of RR^16,54^. *FCGR1A* can differentiate between active and latent tuberculosis, suggesting a major role in the immune response against mycobacterial diseases^34^. Both *CXCL10* and *FCGR1A* showed higher RNA expression during reaction as well as in BL/LL patients compared to TT/BT patients. These markers of innate immunity (*CXCL10)* and infection (*FCGR1A)* thus are increased in two different phases in leprosy associated either with *M. leprae*-specific T cell anergy and high bacterial load (BL/LL) or with a highly inflammatory state (RR). *CXCL10* is associated with Th1 responses occurring during RR^7,13,16^. However, CXCL10*-*producing monocytes are induced during mycobacterial infections^62^, in line with the observed increased expression in BL/LL patients with high bacillary load. Therefore, monitoring transcriptomic changes in an individual is relevant as one marker in various conditions may reflect a different disease process.

In this study 103 target genes for innate and adaptive immune profiling were investigated in view of the immune mediated nature of the pathology of leprosy which strongly correlates with individuals’ immune responses against the bacterium. This selection could limit the discovery of novel genes that could potentially be used as biomarkers for RR. However, in contrast to studies focused at identification of disease mechanisms, for diagnostic purposes only a limited amount of discriminating genes is sufficient, leaving out genes which strongly correlate and hence do not have added value to the signature. Further in-depth transcriptomic analysis of the samples described in this study have been performed by RNASeq in a separate study with the aim to identify additional genes providing increased insight into mechanisms and pathways involved in RR^63^.

Although geographic differences were observed, our study showed that in prospective analyses of 4 different leprosy endemic areas, several genes are associated with onset of reactions at each site. Further analysis of the diagnostic signatures in extended cohorts worldwide need to be performed to validate the performance of the genes signatures.

Transcriptomic analyses have shown that TB^31,32^ disease can be characterized by 16^64^ to as few as 2–4 genes^28^. A gene set signature of reversal reaction consisting of 44 genes was previously described for the Vietnamese population using *M. leprae*-sonicate stimulated whole blood^35^. Their set signature included pro-inflammatory regulator genes such as *CCL2* or *IL1A* in RR. We observed a decreased expression of IL1A after treatment for RR, however we did not find significant differences in patients who later developed RR compared to patients who did not have a reaction. We did not find significant differences either in other genes of that set such as *IL1B*, *IL6*, *IL23A*, *CCL3* or *CCL4*. Another study instead using PBMC of leprosy patients with and without reactions identified a role for complement-associated genes^54^. They also found that *CCL2* and *MARCO*, which are part of our signature biomarker, were differentially expressed in RR patients, as well as an interferon-γ significant upregulation during RR, which is in line with the increased expression of IFN-signaling genes we found longitudinally and our RNAseq data^63^, which shows an enrichment at timepoint of reaction for both IFN- γ and IFN-β pathways.

The here identified biomarker signature is based on unstimulated whole blood and identifies development of RR ≥ 2 months prior to clinical symptoms using a five-gene signature across four leprosy endemic populations on 3 different continents. Unstimulated whole blood and a signature with a small number of markers is more suitable for a point-of-care (POC) use as a field-friendly test. Early diagnosis and treatment of reactions are currently the primary research targets for reducing permanent neuropathy and disability development in future patients. The ability to predict reactions ≥2 months before development of clinical symptoms, using POC diagnostic tests detecting transcriptomic biomarkers for RR, would represent momentous advancements in global leprosy health care. Next steps include prospective evaluation and translation to clinically useful tools that can be implemented by clinicians. The challenge for the academic community and industry is to develop innovative methods to translate multi-transcript signatures into low-cost tests for leprosy diagnostics, suitable for use in health facilities in leprosy endemic areas.

## Supplementary information

Supplementary Material
