# Supplementary material for: Whole blood RNA signatures in leprosy patients identify reversal reactions before clinical onset: a prospective, multicenter study
Source: Sci Rep. 2019 Nov 29;9:17931. doi: 10.1038/s41598-019-54213-y (PMC6884598; doi:10.1038/s41598-019-54213-y)
Supplement: Supplementary file 1 — Supplementary Material [file 41598_2019_54213_MOESM1_ESM.docx]

**Supplementary material**

**Whole blood rna signatures in leprosy patients identify reversal reactions before clinical onset: a prospective, multicenter study**

**Maria Tió-Coma, Anouk van Hooij, Kidist Bobosha, Jolien J. van der Ploeg-van Schip, Sayera Banu, Saraswoti Khadge, Pratibha Thapa, Chhatra B Kunwar, Isabela M. Goulart, Yonas Bekele**, **Deanna A. Hagge, Milton O. Moraes, Rosane M.B. Teles, Roberta Olmo Pinheiro, Erik W. van Zwet, Jelle J. Goeman, Abraham Aseffa, Mariëlle C. Haks, Tom H.M. Ottenhoff, Robert L. Modlin, and Annemieke Geluk**

**Supplementary Results**

**Cross sectional transcriptomic changes in RR patients before and at onset of reactions**

Besides determining whether patients are at risk to develop reactions, biomarkers for RR onset are useful tools for immunomonitoring patients under treatment ^1^. To identify genes indicating onset of RR, we first compared RNA expression profiles of all RR patients before clinical symptoms of reactions (t=0; n=41) versus patients at clinical diagnosis of RR (t=x; n=129). *IL10*, *IL8*, *LRRK2*, *NLRP2*, *CD163*, *IFNG*, *PLP1* and *TNF* were differentially expressed (p-value <0.05). Only *IL10* remained significantly associated with RR after correction for multiple comparisons ^2^ (Table S7; Figure S2).

The effect of treatment was assessed by comparing gene expression levels at clinical onset of RR (t=x; n=129) and after treatment (t=end; n=110). This displayed more variability as significantly higher expression levels were observed at diagnosis of RR for *FCGR1A*, *IL1A* and *IL21*, whereas for *IL10*, *PLP1* and *FLCN1* the opposite was found (Table S8; Figure S3). In contrast, no significant differences in RNA expression levels were observed for any of these genes in non-endemic controls (NEC) following longitudinal analyses (data not shown).

**Supplementary tables**

**Supplementary Table S1:** *Target genes* tested in dcRT-MLPA.*

| *AIRE* |  | *CSF2* |  | ***IFI44*** |  | *IL17A* |  | *NLRP10* |  | *TLR2* |
| --- | --- | --- | --- | --- | --- | --- | --- | --- | --- | --- |
| ***ASAP1*** |  | *CSF3* |  | ***IFI44L*** |  | *IL18* |  | *NLRP11* |  | *TLR3* |
| ***BLR1*** |  | *CTLA4* |  | ***IFI6*** |  | *IL21* |  | *NLRP12* |  | *TLR4* |
| ***BMP6*** |  | ***CX3CL1*** |  | ***IFIH1*** |  | *IL22RA1* |  | *NLRP13* |  | *TLR5* |
| *CAMTA1* |  | *CXCL10* |  | ***IFIT2*** |  | *IL23A* |  | *NOD2* |  | *TLR6* |
| *CCL2* |  | *CXCL13* |  | ***IFIT3*** |  | *IL32* |  | ***OAS1*** |  | *TLR7* |
| *CCL3* |  | ***EGF*** |  | ***IFIT5*** |  | ***INDO*** |  | ***OAS2*** |  | *TLR8* |
| *CCL4* |  | ***ERBB2*** |  | ***IFITM3*** |  | ***IRF7*** |  | ***OAS3*** |  | *TLR9* |
| ***CCL11*** |  | *FCGR1A* |  | *IFNG* |  | ***KIF1B*** |  | ***PACRGv1*** |  | *TLR10* |
| *CCL22* |  | *FCGR1B* |  | *IGF1* |  | *LAG3* |  | ***PARK2v1,2*** |  | *TNF* |
| *CCR6* |  | *FLCN1* |  | *IL1A* |  | *LIPE* |  | ***PHEX*** |  | *TNFRSF18* |
| *CCR7* |  | *FOXP3* |  | *IL1B* |  | ***LRRK2*** |  | *PLP1* |  | ***TNIP1*** |
| ***CD14*** |  | *GATA3* |  | *IL10* |  | ***LTA4H*** |  | *PRF1* |  | *TWIST1* |
| *CD163* |  | ***GBP1*** |  | *IL2* |  | ***LYN*** |  | *PTPRCv1* |  | ***VDR*** |
| *CD19* |  | ***GBP2*** |  | *IL2RA* |  | *MARCO* |  | *PTPRCv2* |  | *VEGFA* |
| *CD209* |  | ***GBP5*** |  | *IL4* |  | *MBP* |  | *RORC* |  | *ZNF532* |
| ***CD274*** |  | *GNLY* |  | *IL5* |  | *MMP2* |  | ***SLAMF7*** |  |  |
| *CD36* |  | *GZMA* |  | *IL6* |  | *MSR1* |  | ***SOCS1*** |  |  |
| *CD3E* |  | *GZMB* |  | *IL7* |  | *NEDD4L* |  | ***STAT1*** |  |  |
| *CD4* |  | ***HCK*** |  | ***IL7R*** |  | *NLRP1* |  | ***STAT2*** |  |  |
| *CD8A* |  | *HDAC1* |  | *IL8* |  | *NLRP2* |  | ***TAP1*** |  |  |
| *CD46* |  | *HDAC2* |  | *IL9* |  | *NLRP3* |  | ***TAP2*** |  |  |
| *CD68* |  | ***HPRT*** |  | *IL12A* |  | *NLRP4* |  | *TBX21* |  |  |
| *CFB* |  | ***IFI16*** |  | *IL13* |  | *NLRP6* |  | *TGFB* |  |  |
| *CFH* |  | ***IFI35*** |  | *IL15* |  | *NLRP7* |  | *TLR1* |  |  |

**Gene expression profiling was performed on 141 target genes (for innate and adaptive immunity or leprosy susceptibility) plus household genes (ABR, B2M, GAPDH, GUSB) using three distinct dcRT-MLPA gene sets. Genes depicted in bold (n=38) were used only for longitudinal samples of reactional patients. Genes depicted in bold underlined (n=7) were included in the analysis based on their reported role in susceptibility to develop leprosy ^3-9^.*

**Supplementary Table S2:** *Target genes differentially expressed between endemic controls (n=200) vs leprosy patients (n= 359).**

| **Gene** | **Raw p-value** | **Adjusted p-value**** | **Direction***** |
| --- | --- | --- | --- |
| *CD3E* | 2.37e-12 | 2.35e-10 | down |
| *GZMA* | 3.84e-11 | 1.90e-09 | down |
| *FCGR1A* | 6.22e-10 | 2.05e-08 | up |
| *IL6* | 1.78e-09 | 4.40e-08 | up |
| *IL15* | 3.79e-09 | 7.50e-08 | up |
| *CCR6* | 2.24e-08 | 3.69e-07 | down |
| *TLR3* | 8.64e-08 | 1.22e-06 | down |
| *GATA3* | 3.99e-06 | 4.94e-05 | down |
| *CCL2* | 6.88e-06 | 7.57e-05 | down |
| *CCR7* | 1.07e-05 | 1.06e-04 | down |
| *CTLA4* | 1.63e-05 | 1.47e-04 | down |
| *PACRGv1* | 2.45e-05 | 2.02e-04 | up |
| *NLRP13* | 4.98e-05 | 3.79e-04 | down |
| *TLR5* | 7.03e-05 | 4.97e-04 | up |
| *HDAC1* | 8.78e-05 | 5.80e-04 | down |
| *CFB* | 1.57e-04 | 9.71e-04 | up |
| *CD8A* | 2.34e-04 | 1.36e-03 | down |
| *IL9* | 3.23e-04 | 1.78e-03 | down |
| *IL2* | 3.94e-04 | 2.05e-03 | up |
| *CD163* | 5.77e-04 | 2.86e-03 | down |
| *CD4* | 6.39e-04 | 3.01e-03 | down |
| *VDR* | 7.29e-04 | 3.28e-03 | up |
| *IFNG* | 7.96e-04 | 3.43e-03 | down |
| *IL32* | 1.13e-03 | 4.65e-03 | down |
| *NLRP1* | 2.03e-03 | 8.02e-03 | down |
| *TLR4* | 2.11e-03 | 8.05e-03 | up |
| *CCL4* | 2.47e-03 | 9.05e-03 | up |
| *TBX21* | 3.39e-03 | 1.17e-02 | down |
| *TLR10* | 3.44e-03 | 1.17e-02 | up |
| *IL1B* | 3.54e-03 | 1.17e-02 | up |
| *FOXP3* | 8.27e-03 | 2.64e-02 | down |
| *NLRP2* | 8.55e-03 | 2.65e-02 | down |
| *LRRK2* | 1.21e-02 | 3.54e-02 | up |
| *CD209* | 1.22e-02 | 3.54e-02 | down |
| *MARCO* | 1.29e-02 | 3.64e-02 | down |
| *TLR2* | 1.76e-02 | 4.83e-02 | down |

**Cross-sectional RNA expression analysis of 103 target genes (Table S1) in ex vivo whole blood samples from leprosy patients at diagnosis (t=0) in the absence of any clinical signs of reactions and healthy controls from the same living area.*

***Differentially expressed genes after correction for multiple comparisons using the method of Benjamini and Hochberg ^2^ are shown.*

****Direction is up when gene expression is upregulated in leprosy patients.*

**Supplementary Table S3:** *Target genes differentially expressed between healthy household contacts (n=83) vs leprosy patients (n= 359).**

| **Gene** | **Raw p-value** | **Adjusted p-value**** | **Direction***** |
| --- | --- | --- | --- |
| *TGFB* | 7.76e-07 | 7.68e-05 | down |
| *IL15* | 4.11e-06 | 2.03e-04 | up |
| *LAG3* | 2.77e-05 | 9.14e-04 | down |
| *CXCL13* | 4.00e-05 | 9.90e-04 | down |
| *GATA3* | 1.62e-04 | 3.20e-03 | down |
| *TLR1* | 1.42e-03 | 2.34e-02 | up |
| *MBP* | 1.87e-03 | 2.47e-02 | up |
| *CTLA4* | 2.00e-03 | 2.47e-02 | down |
| *LRRK2* | 2.69e-03 | 2.56e-02 | up |
| *IL6* | 2.72e-03 | 2.56e-02 | up |
| *CD3E* | 3.10e-03 | 2.56e-02 | down |
| *CAMTA* | 3.11e-03 | 2.56e-02 | down |
| *TLR4* | 3.36e-03 | 2.56e-02 | up |
| *PACRGv1* | 3.79e-03 | 2.68e-02 | up |
| *MSR1* | 5.52e-03 | 3.64e-02 | up |
| *FCGR1A* | 6.87e-03 | 4.25e-02 | up |

**Cross-sectional RNA expression analysis of 103 target genes (Table S1) in ex vivo whole blood samples from leprosy patients at diagnosis (t=0) in the absence of any clinical signs of reactions and healthy household contacts from the same living area.*

***Differentially expressed genes after correction for multiple comparisons using the method of Benjamini and Hochberg ^2^ are shown.*

****Direction is up when gene expression is upregulated in leprosy patients.*

**Supplementary Table S4:** *Relevant pathways identified* that contain multiple significantly different expressed genes between leprosy patients (n=359) and endemic controls (n=200) or leprosy patients and healthy household contacts (n=83).*

| **Pathway** | **Number of genes** | **Significant genes identified in pathway**** |
| --- | --- | --- |
| **Leprosy patients vs endemic controls** |  |  |
| Communication between Innate and Adaptive Immune Cells | 14 | CCL4, CCR7, CD4, CD8A, IFNG, IL2, IL6, IL15, IL1B, TLR2, TLR3, TLR4, TLR5, TLR10 |
| Role of Macrophages, Fibroblasts and Endothelial Cells in Rheumatoid Arthritis | 11 | CCL2, FCGR1A, IL6, IL15, IL32, IL1B, TLR2, TLR3, TLR4, TLR5, TLR10 |
| Altered T Cell and B Cell Signaling in Rheumatoid Arthritis | 10 | IFNG, IL2, IL6, IL15, IL1B, TLR2, TLR3, TLR4, TLR5, TLR10 |
| Neuroinflammation Signaling Pathway | 9 | CCL2, IFNG, IL6, IL1B, TLR2, TLR3, TLR4, TLR5, TLR10 |
| TREM1 Signaling | 9 | CCL2, IL6, IL1B, NLRP2, TLR2, TLR3, TLR4, TLR5, TLR10 |
| Dendritic Cell Maturation | 9 | CCR7, FCGR1A, IL6, IL15, IL32, IL1B, TLR2, TLR3, TLR4 |
| Th1 and Th2 Activation Pathway | 9 | CD4, CD3E, CD8A, GATA3, IFNG, IL2, IL6, IL9, TBX21 |
| **Leprosy patients vs household contacts** |  |  |
| Altered T Cell and B Cell Signaling in Rheumatoid Arthritis | 6 | CXCL13, IL6, IL15, TGFB1, TLR1, TLR4 |
| Role of Macrophages, Fibroblasts and Endothelial Cells in Rheumatoid Arthritis | 6 | FCGR1A, IL6, IL15, TGFB1, TLR1, TLR4 |

**Pathways identified using Ingenuity Pathway Analysis (Qiagen, Hildern, Germany) based on the genes identified in Tables S2-S3.*

***Genes involved in the pathway with a differential expression (p-value <0.05) between leprosy patients and endemic controls or leprosy patients and healthy household contacts.*

**Supplementary Table S5:** *Target genes differentially expressed between BL/LL (n=228) vs TT/BT leprosy patients (n=131).**

| **Gene** | **Raw p-value** | **Adjusted p-value**** | **Direction***** |
| --- | --- | --- | --- |
| *FCGR1A* | 1.37e-05 | 1.36e-03 | down |
| *CD46* | 1.55e-04 | 7.66e-03 | down |
| *TLR4* | 7.86e-04 | 1.65e-02 | down |
| *HDAC2* | 7.98e-04 | 1.65e-02 | down |
| *IL2* | 8.35e-04 | 1.65e-02 | up |
| *CXCL10* | 1.14e-03 | 1.88e-02 | down |
| *TLR6* | 2.24e-03 | 2.77e-02 | up |

** Cross-sectional gene expression analysis: RNA expression levels were* *determined of BL/LL and TT/BT patients at diagnosis of leprosy (t=0) in the absence of any clinical signs of reactions, at least two months before diagnosis of RR in case of reactional patients.*

***Differentially expressed genes after correction for multiple comparisons using the method of Benjamini and Hochberg ^2^ are shown.*

****Direction is up when gene expression is higher in TT/BT patients compared to BL/LL patients.*

**Supplementary Table S6:** *Target genes differentially expressed between endemic controls (n=200) vs healthy household contacts (n=83).**

| **Gene** | **Raw p-value** | **Adjusted p-value**** | **Direction***** |
| --- | --- | --- | --- |
| *CCR6* | 1.17e-06 | 1.16e-04 | down |
| *HDAC2* | 6.16e-04 | 2.25e-02 | down |
| *FOXP3* | 8.61e-04 | 2.25e-02 | up |
| *TLR7* | 1.11e-03 | 2.25e-02 | down |
| *TGFB* | 1.24e-03 | 2.25e-02 | up |
| *TLR1* | 1.36e-03 | 2.25e-02 | down |
| *GZMA* | 1.59e-03 | 2.25e-02 | down |
| *IL22RA1* | 2.30e-03 | 2.85e-02 | down |
| *PTPRCv2* | 3.09e-03 | 3.40e-02 | down |
| *CCL3* | 4.25e-03 | 4.04e-02 | up |

** Cross-sectional gene expression analysis: RNA expression levels were* *determined for healthy household contacts of BL/LL patients and endemic controls. If data from several time points were available for HHC, only data of the first visit was included in the analysis.*

***Differentially expressed genes after correction for multiple comparisons using the method of Benjamini and Hochberg ^2^ are shown.*

****Direction is up when gene expression is higher in healthy household contacts compared to endemic controls.*

**Supplementary Table S7:** *Target genes differentially expressed between patients with RR (n=30) vs non-reactional BL/LL patients at t=0 (n=184).**

| **Gene** | **Raw p-value** | **Adjusted p-value**** | **Direction***** |
| --- | --- | --- | --- |
| *IL15* | 9.29e-06 | 9.10e-04 | up |
| *TLR2* | 3.64e-05 | 1.79e-03 | up |
| *IL2* | 5.74e-05 | 1.88e-03 | up |
| *CTLA4* | 2.43e-04 | 5.94e-03 | down |
| *MARCO* | 5.68e-04 | 1.00e-02 | up |
| *CCL2* | 6.14e-04 | 1.00e-02 | up |
| *TLR6* | 8.95e-04 | 1.15e-02 | up |
| *TLR10* | 9.37e-04 | 1.15e-02 | up |
| *IL18* | 1.93e-03 | 2.10e-02 | up |
| *PHEX* | 4.04e-03 | 3.96e-02 | up |
| *GATA3* | 4.94e-03 | 4.40e-02 | down |

**Cross-sectional gene expression analysis: RNA expression was* *determined for all patients at diagnosis of leprosy (t=0) in the absence of any clinical signs of reactions, at least two months before diagnosis of RR in case of reactional patients.*

***Differentially expressed genes after correction for multiple comparisons using the method of Benjamini and Hochberg ^2^ are shown.*

****Direction is up when gene expression is upregulated in reactional patients.*

**Supplementary Table S8:** *Gene expression for patients with RR at t=0 (n=41) vs t=x (n=129).**

| **Gene** | **Raw p-value** | **Adjusted p-value**** | **Direction***** |
| --- | --- | --- | --- |
| ***IL10*** | 2.36e-04 | 2.25e-02 | up |
| *IL8* | 3.00e-03 | 1.43e-01*^¶^* | up |
| *LRRK2* | 9.78e-03 | 3.10e-01*^¶^* | up |
| *NLRP2* | 1.31e-02 | 3.12e-01*^¶^* | up |
| *CD163* | 1.64e-02 | 3.12e-01*^¶^* | up |
| *IFNG* | 2.97e-02 | 4.38e-01*^¶^* | down |
| *PLP1* | 3.48e-02 | 4.38e-01*^¶^* | up |
| *TNF* | 3.68e-02 | 4.38e-01*^¶^* | up |

**Longitudinal gene expression analysis: RNA expression for each patient with RR was* *determined at diagnosis of leprosy (t=0) in the absence of any clinical signs of reactions, at least two months before diagnosis of RR and compared to RNA expression at RR diagnosis before steroids (t=x);*

***Differentially expressed genes after correction for multiple comparisons using the method of Benjamini and Hochberg ^2^ are shown.*

****Direction is up when gene expression is upregulated before reaction (t=0).*

*^¶^ not significant; indicated for reference purposes only.*

**Supplementary Table S9:** *Gene expression for patients with RR at t=Rxn (n=129) vs t=end (n=110).**

| **Gene** | **Raw p-value** | **Adjusted p-value**** | **Direction***** |
| --- | --- | --- | --- |
| *FCGR1A* | 2.23e-04 | 1.98e-02 | down |
| *IL1A* | 5.80e-04 | 1.98e-02 | down |
| *IL10* | 5.89e-04 | 1.98e-02 | up |
| *IL21* | 1.04e-03 | 2.21e-02 | down |
| *FLCN1* | 1.06e-03 | 2.21e-02 | up |
| *PLP1* | 2.37e-03 | 3.99e-02 | up |

**Longitudinal gene expression analysis: RNA expression for each patient with RR was* *determined at RR diagnosis before steroids (t=x) and compared to RNA expression after MDT and at least one month after end of steroids, in the absence of reactions (t=end).*

***Differentially expressed genes after correction for multiple comparisons using the method of Benjamini and Hochberg ^2^ are shown.*

****Direction is up when gene expression is upregulated at reaction (t=x).*

**Supplementary figures**

**
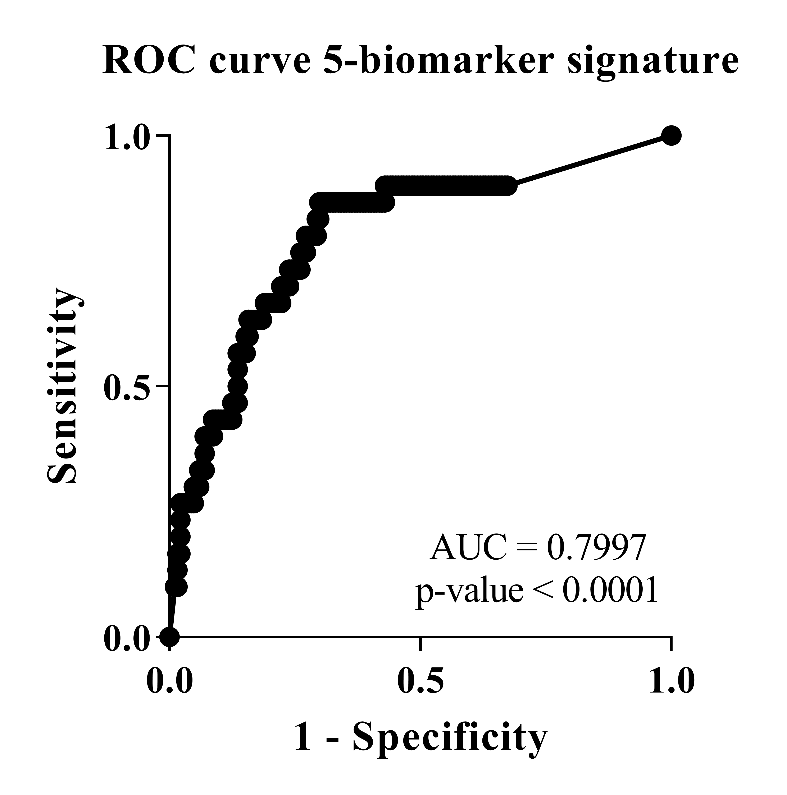
**

**Supplementary Figure S1: AUC 5-biomarker signature to predict RR.**

Performance of a 5-biomarker signature defined by global test and composed of gene expression data of *CCL2*, *CD8A*, *IL2*, *IL15* and *MARCO* to predict RR. And out-of-bag approach was used to assess the risk of developing RR in which a sample was excluded from the training set on each iteration. Receiver operator characteristics (ROC) curve is shown for the accuracy of the biomarker signature. The true positive rate (sensitivity) is plotted against the false positive rate (1-specificity). AUC: area under the curve.

**
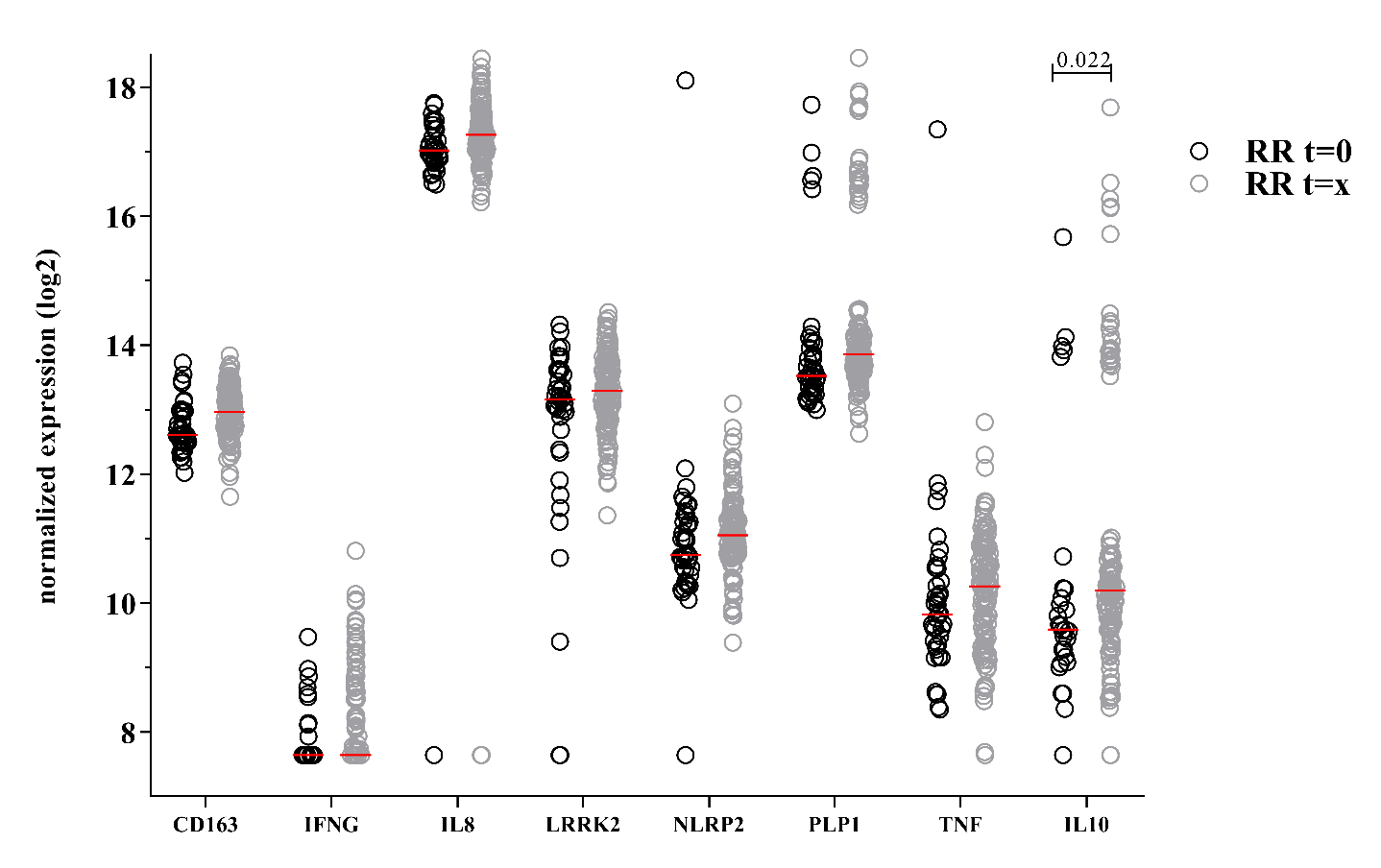
**

**Supplementary Figure S2:** Difference in gene expression in cross sectional samples between time points: before RR and at RR.

Gene expression data obtained by dcRT-MLPA of RNA isolated from unstimulated whole blood of RR patients from Bangladesh, Brazil, Ethiopia and Nepal at t=0 (n= 41) and at clinical onset of RR (n=129, t=x). Log2-transformations of peak areas (normalized to the housekeeping gene *GAPDH*) of genes that were significantly different are shown on the *y*-axis. Raw p-values were calculated using the Mann–Whitney test and adjusted for multiple comparisons using the Benjamini-Hochberg correction ^2^.

**
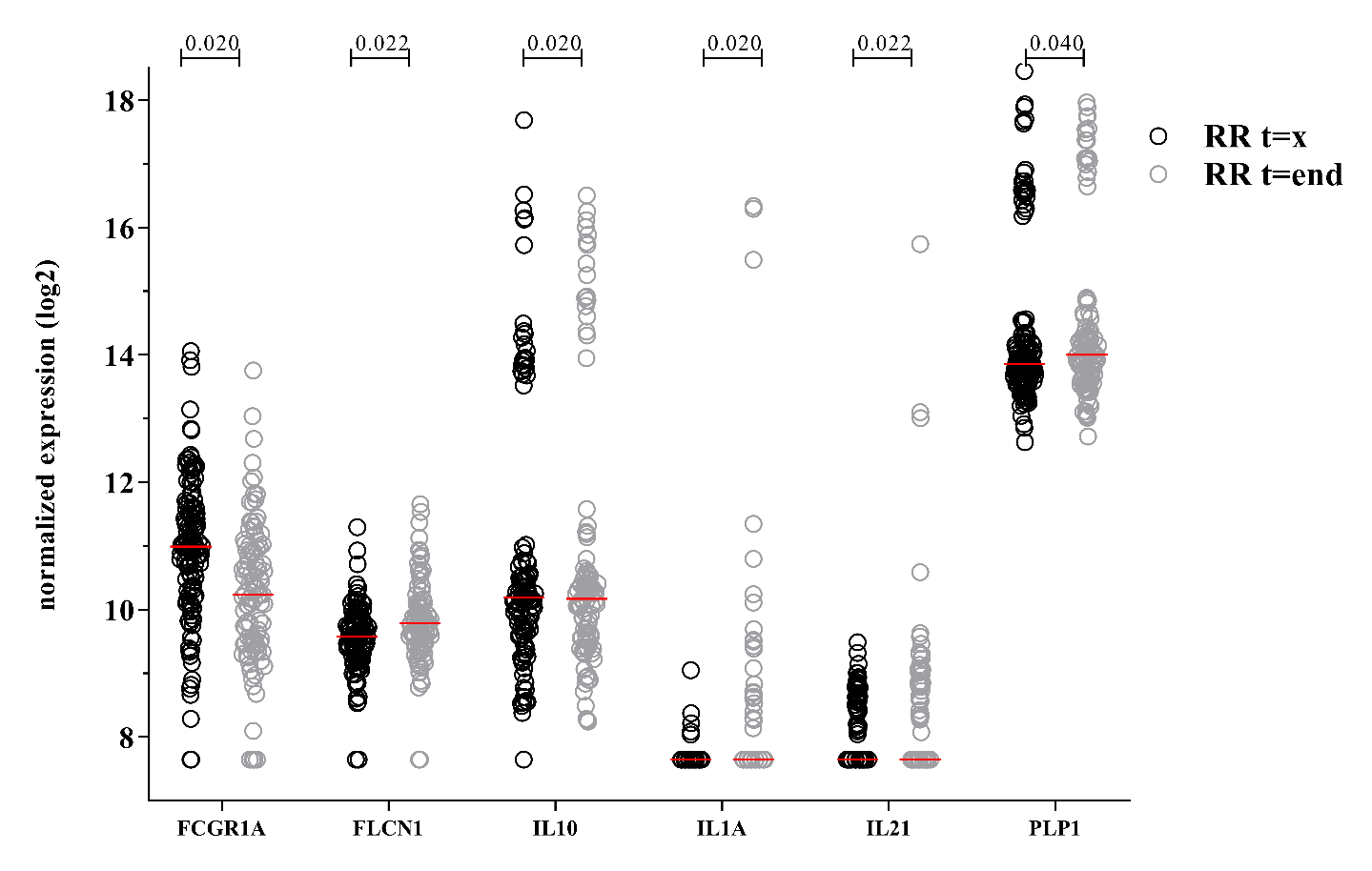
**

**Supplementary Figure S3:** Difference in gene expression in cross sectional samples between time points: at RR and after treatment.

Gene expression data obtained by dcRT-MLPA of RNA isolated from unstimulated whole blood of RR patients from Bangladesh, Brazil, Ethiopia and Nepal at clinical onset of RR (n=129, t=x) and after treatment (n=110, t=end). Log2-transformations of peak areas (normalized to the housekeeping gene *GAPDH*) of genes that were significantly different are shown on the *y*-axis. Raw p-values were calculated using the Mann–Whitney test and adjusted for multiple comparisons using the Benjamini-Hochberg correction ^2^.

**
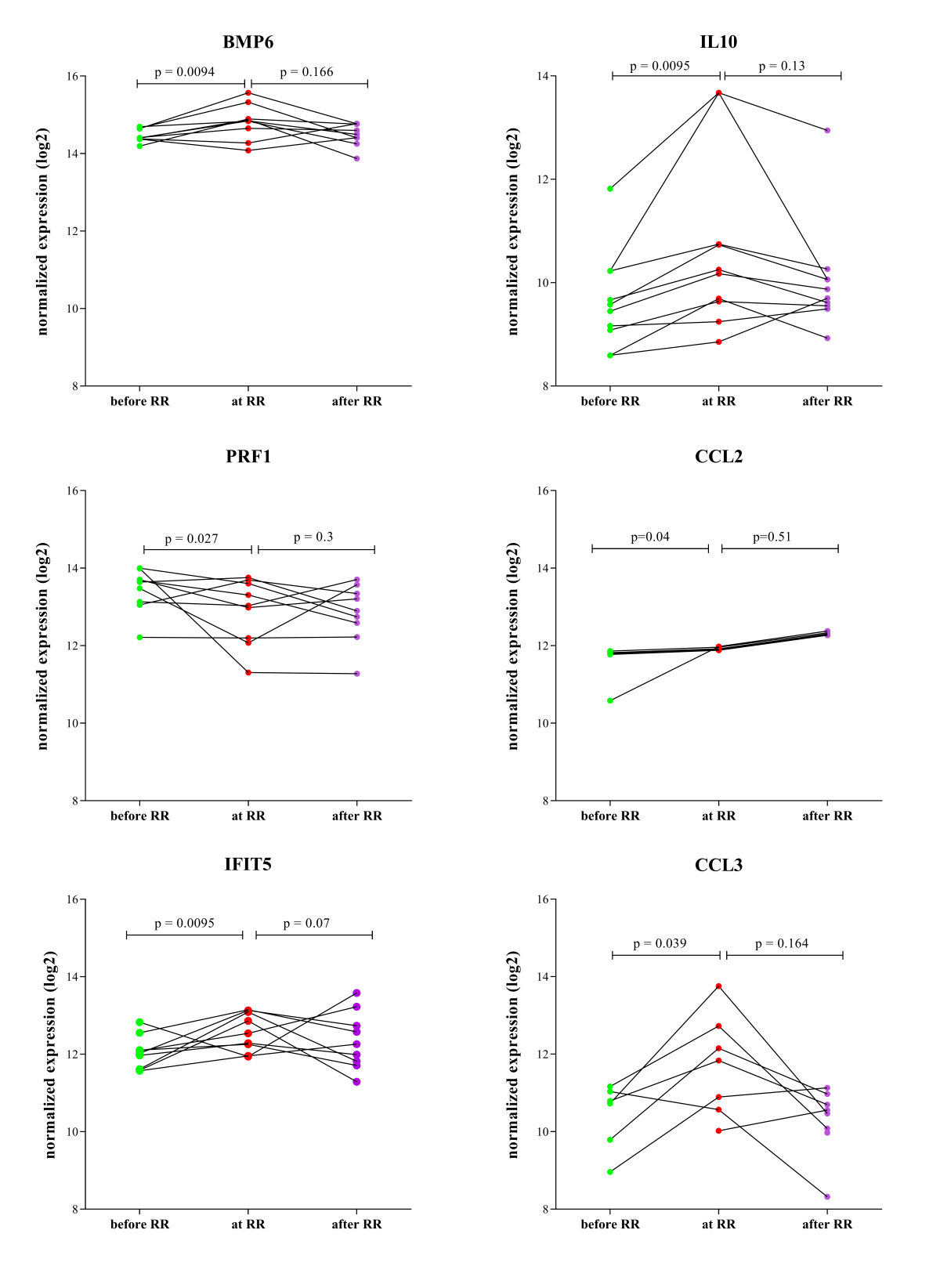
**

**Supplementary Figure S4:** Set of genes showing significant differences in expression levels during longitudinal follow-up before RR and at RR.

Gene expression was assessed by dcRT-MLPA on *ex vivo* RNA of 10 leprosy patients who developed RR during this study. Blood was analyzed at 3 time points: in the absence of any clinical signs of reactions and at least two months before RR (t=0, in green), at RR diagnosis before steroids (t=x, in red) or after MDT and at least one month after end of steroids, in the absence of reactions (t=end, in purple). Log2-transformations of peak areas (normalized for *GAPDH* expression) are shown on the *y*-axis. Wilcoxon signed-rank test was performed. Significant differences (p-value <0.05) between gene expression before RR and at RR are indicated.

**
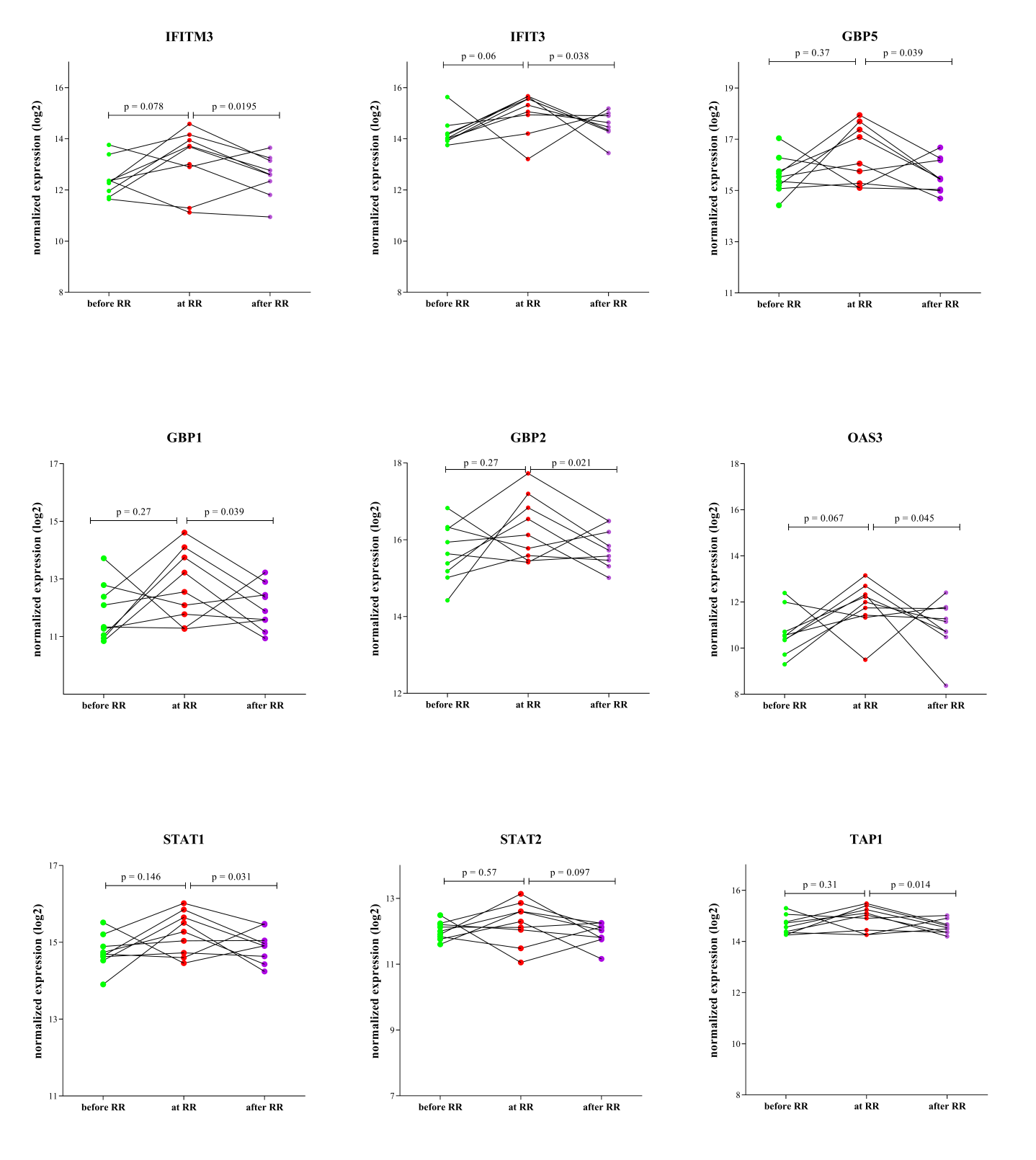
**

**Supplementary Figure S5:** Set of genes showing significant differences in expression levels during longitudinal follow-up between RR and after RR.

Gene expression levels were assessed by dcRT-MLPA on *ex vivo* RNA of 10 leprosy patients who developed RR during this study. Blood was analyzed at 3 time points: in the absence of any clinical signs of reactions and at least two months before RR (t=0, in green), at RR diagnosis before steroids (t=x, in red) or after MDT and at least one month after end of steroids, in the absence of reactions (t=end, in purple). Log2-transformations of peak areas (normalized for *GAPDH* expression) are shown on the *y*-axis. Wilcoxon signed-rank test was performed. Significant differences (p-value <0.05) between gene expression before RR and at RR are indicated.


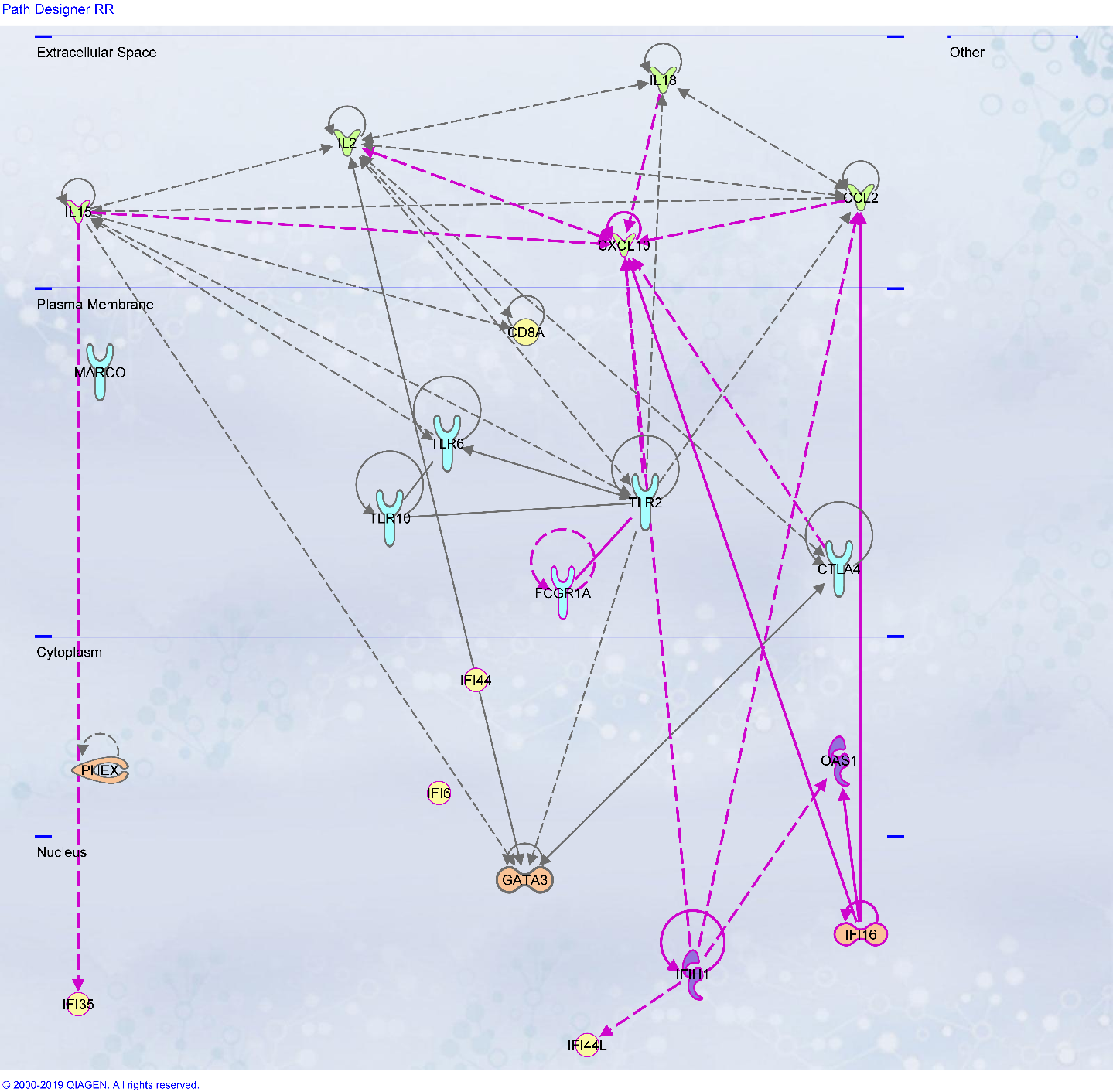


**Supplementary Figure S6:** Relationship of the genes identified as predictive CoR in RR and differently expressed longitudinally.

Data were analyzed by Ingenuity Pathway Analysis^10^ (QIAGEN Inc., <https://www.qiagenbioinformatics.com/products/ingenuitypathway-analysis>) showing the connections between the genes identified as predictive CoR for RR before clinical symptoms and genes with a significantly different expression in RR patients longitudinally. Genes identified in the longitudinal analysis are depicted with pink lines and genes identified as predictive CoR for RR in gray.

**References**

1 Geluk, A. *et al.* Longitudinal immune responses and gene expression profiles in type 1 leprosy reactions. *Journal of clinical immunology* **34**, 245-255 (2014).

2 Benjamini, Y. & Hochberg, Y. Controlling the False Discovery Rate - a Practical and Powerful Approach to Multiple Testing. *J R Statist Soc B* **57**, 289-300 (1995).

3 Tapinos, N., Ohnishi, M. & Rambukkana, A. ErbB2 receptor tyrosine kinase signaling mediates early demyelination induced by leprosy bacilli. *Nat. Med* **12**, 961-966 (2006).

4 Mira, M. T. *et al.* Susceptibility to leprosy is associated with PARK2 and PACRG. *Nature* **427**, 636-640 (2004).

5 Rodrigues, L. S. *et al.* Circulating levels of insulin-like growth factor-I (IGF-I) correlate with disease status in leprosy. *BMC infectious diseases* **11**, 339 (2011).

6 Cardoso, C. C., Pereira, A. C., de Sales Marques, C. & Moraes, M. O. Leprosy susceptibility: genetic variations regulate innate and adaptive immunity, and disease outcome. *Future microbiology* **6**, 533-549 (2011).

7 Tobin, D. M. *et al.* The lta4h locus modulates susceptibility to mycobacterial infection in zebrafish and humans. *Cell* **140**, 717-730 (2010).

8 Silva, S. R. *et al.* Downregulation of PHEX in multibacillary leprosy patients: observational cross-sectional study. *Journal of translational medicine* **13**, 296 (2015).

9 Wang, D. *et al.* Association of the LRRK2 genetic polymorphisms with leprosy in Han Chinese from Southwest China. *Genes Immun* **16**, 112-119 (2015).

10 Kramer, A., Green, J., Pollard, J., Jr. & Tugendreich, S. Causal analysis approaches in Ingenuity Pathway Analysis. *Bioinformatics* **30**, 523-530 (2014).
